# SIRT3 Regulates the ROS-FPR1/HIF-1α Axis under Hypoxic Conditions to Influence Lung Cancer Progression

**DOI:** 10.1007/s12013-023-01180-x

**Published:** 2023-09-25

**Authors:** Bo Huang, Jie Ding, HongRong Guo, HongJuan Wang, JianQun Xu, Quan Zheng, LiJun Zhou

**Affiliations:** 1 Wuhan Third Hospital/Tongren Hospital of Wuhan University, Wuhan East Lake High-Tech Development Zone Jiufeng Street Center City Community Health Service Center, Wuhan, 430074 Hubei China; 2 Wuhan Third Hospital/Tongren Hospital of Wuhan University, Wuhan, 430074 Hubei China

**Keywords:** Lung cancer, SIRT3, ROS-FPR1/HIF-1α axis, Proliferation, Inflammatory response

## Abstract

Hypoxia-inducible factor (HIF-1α) is a therapeutic target in lung cancer, and the deacetylase sirtuin 3 (SIRT3) is closely associated with tumorigenesis. Formyl peptide receptor 1 (FPR1) is involved in a wide range of physiopathological processes in various tumor cells. We explored whether SIRT3 affects the development of lung cancer by regulating the reactive oxygen species (ROS)-FPR1/HIF-1α axis under hypoxic conditions. The effects of SIRT3 overexpression on the levels of FPR1, HIF-1α, ROS, inflammatory factors, and cell proliferation and migration in A549 cells under hypoxic conditions were assessed in combination with the FPR1 inhibitor. BALB/c nude mice were subcutaneously injected with cancer cells transfected/untransfected with SIRT3 overexpressing lentiviral vectors. Immunohistochemistry and enzyme-linked immunosorbent assay were performed to detect SIRT3 expression and the expression levels of IL-1β, TNF-α, and IL-6, respectively, in tumor tissues. Cell proliferation, invasion, migration, and IL-1β, TNF-α, IL-6, and ROS levels were significantly higher in the Hypoxia group than in the Control group. Moreover, the mRNA and protein expression levels of SIRT3 were significantly down-regulated, whereas they were significantly up-regulated for FPR1 and HIF-1α. In contrast, SIRT3 overexpression in a hypoxic environment inhibited cell proliferation, invasion, and migration, decreased IL-1β, TNF-α, IL-6, and ROS levels, up-regulated the mRNA and protein expression levels of SIRT3, and down-regulated the mRNA and protein expression levels of FPR1 and HIF-1α. In addition, we found the same results in tumorigenic experiments in nude mice. SIRT3 in hypoxic environments may affect tumor cell proliferation, invasion, migration, and inflammation levels via the ROS-FPR1/HIF-1α axis, thereby inhibiting tumor cell development.

## Introduction

Patients with lung cancer are typically not diagnosed until the cancer is advanced, which results in an increased mortality rate [1]. The current treatment for lung cancer is primarily based on chemotherapy with cisplatin (CDDP, cis-diamminedichloroplatinum); however, chemotherapy drugs have adverse side effects, due to their lack of specificity. Therefore, detailed pathogenesis, effective early detection, and new therapeutic targets must be further investigated [2]. Hypoxia-inducible factor (HIF-1α) is a therapeutic target in lung cancer that monitors how cells respond to oxygen levels in solid tumors. In patients with gastric cancer, chronic hypoxia results in HIF-1α activation, which is highly associated with an aggressive tumor phenotype and poor prognosis [3].

Mitochondrial dysfunction plays important roles in apoptosis, proliferation, and the bioenergetic reprogramming of cancer cells [4]. The deacetylase sirtuin 3 (SIRT3) is an important factor in maintaining mitochondrial function and regulates mitochondrial respiration and antioxidant stress enzymes [5]. Moreover, SIRT3 could increase the sensitivity of small cell lung cancer to cisplatin via apoptosis [6]. The deletion of SIRT3 enhances the hypoxic activation of HIF-1α, and hypoxia induces the production of reactive oxygen species (ROS) in the mitochondria; therefore, the alteration of ROS levels may be the primary mechanism by which SIRT3 regulates HIF-1α [7]. However, whether SIRT3 can affect oxidative stress and HIF-1α expression in lung cancer cells by mediating ROS under hypoxic conditions has not been reported.

Formyl peptide receptor 1 (FPR1) is a pattern-recognition receptor with important roles in innate immunity, wound repair, and angiogenesis [8]. Our previous study found that the inhibition of FPR1 prevented lung adenocarcinoma cell invasion and migration [9]. FPR1 is highly expressed in the blood of non-small cell lung cancer patients and can be used as a single biomarker for the detection of lung cancer [10]. In addition, FPR1 can regulate oxidative stress via nicotinamide adenine dinucleotide phosphate oxidase-dependent ROS production. FPR1 can promote the progression of different cardiovascular diseases by activating the MAPKs pathway, generating ROS, and releasing pro-inflammatory cytokines (i.e., IL-1β, IL-6, TNF-α) to promote the inflammatory state [11]. However, the roles of FPR1 in relation to oxidative stress and inflammation in hypoxia-induced lung cancer cells are unclear.

In this study, we evaluated the effect of SIRT3 overexpression on A549 lung cancer cells under hypoxic conditions using in vitro experiments. Lung adenocarcinoma cells that were transfected/untransfected with SIRT3 overexpression lentiviral vectors were injected subcutaneously to assess the effect of SIRT3 overexpression on the ROS-FPR1/HIF-1α axis under hypoxic conditions. The aim was to investigate whether SIRT3 affected lung cancer development under hypoxic conditions via regulating the ROS-FPR1/ HIF-1α axis.

## Materials and Methods

### Cell Culture and Grouping

A549 cells were acquired from the Shanghai Cell Bank, Chinese Academy of Sciences (Shanghai, China) and removed from liquid nitrogen. First, 4 mL of complete medium (F12 K + 10% fetal bovine serum (FBS)) was added to the cells, and the samples were centrifuged at 400 × *g* for 3 min. Cells were resuspended in 1 mL of medium, followed by the addition of 4 mL of complete medium. The samples were then incubated at 37 °C in a 5% CO_2_ incubator. The cells were divided into the following groups: Control (incubated in normoxia (21% O_2_) for 24 h), Hypoxia (incubated in hypoxia (5–8% O_2_) for 24 h), SIRT3 mimics (transfected with SIRT3 overexpression vector + hypoxia (5–8% O_2_) for 24 h), Vector (transfected with SIRT3 overexpression empty vector + hypoxia (5–8% O_2_) for 24 h), SIRT3 mimics+Ant FPRl (1 μM Boc2 treatment for 24 h + hypoxia (5–8% O_2_) for 24 h), Vector+Ant FPRl (transfected with SIRT3 overexpression empty vector + hypoxia (5–8% O_2_) for 24 h + 1 μM Boc2 treatment for 24 h), and Ant FPRl (1 μM Boc2 treatment for 24 h) groups.

### Transwell

Transwell upper chambers were pre-coated with Matrigel (BD, USA), and 2 × 10^5^ cells were inoculated in the upper chamber containing serum-free RPMI 1640 medium. RPMI 1640 medium containing 10% FBS was added to the lower chamber. Following 48 h of incubation, the uninvaded cells on the lower surface of the upper chamber were removed with cotton swabs, and the invaded cells were fixed with 4% paraformaldehyde and stained with 0.1% crystal violet. Cell invasion was observed under a light microscope.

### Cell Scratching

A549 cells were inoculated (1 × 10^6^ cells/well) and cultured at 37 °C with 5% CO_2_. When the cell confluence attained 100%, the cell layer was scratched using a 200 µL pipette tip, and the RPMI 1640 medium containing 10% FBS was replaced with RPMI 1640 medium without FBS. The scratch healing images of the cells at 0 and 24 h were observed using light microscopy.

### Western Blot

Total protein in the cells was extracted using radioimmunoprecipitation assay lysis buffer (Solarbio, China). Following protein quantification, electrophoretic separation, membrane transfer, and closure, polyvinylidene difluoride membranes were incubated with the 1:1000 dilutions of primary antibodies MMP-2, MMP9, HIF-1α, SIRT3, FPR1, E- cadherin, and GAPDH (all from Bioswamp, China). The samples were incubated overnight at 4 °C. A secondary antibody (1:20,000) was added, and the samples were incubated at room temperature for 2 h. An enhanced chemiluminescence reagent was added to observe protein color development.

### qRT-PCR

Total RNA was extracted using the Trizol reagent (Ambion, USA). RNA was reverse transcribed into cDNA and then used as a template for fluorescent qPCR. The gene expression levels were calculated using the 2^−ΔΔCt^ method with GAPDH as the internal reference. PCR primers were synthesized by Wuhan Tianyi Huayu Gene Technology Co. The primer sequences are presented in Table 1.

**Table 1:** Primer sequences

Primer	Sequence	Size (bp)
HIF-1α-F	AAGTGTACCCTAACTAGCCG	160
HIF-1α-R	CACAAATCAGCACCAAGC	
SIRT3-F	GCATCCCTGCCTCAAAG	100
SIRT3-R	TCAGCCCGAATGTCCTC	
FPR1-F	AAACGGGGACAGTAGCC	151
FPR1-R	CTGACAGCAACGATGGAC	
GAPHD-F	GGGAAACTGTGGCGTGAT	299
GAPDH-R	GAGTGGGTGTCGCTGTTGA	

### ROS

2’,7’-Dichlorodihydrofluorescein diacetate (DCFH-DA) was diluted with serum-free culture medium (1:1000) to a final concentration of 10 μmol/L. Cells were collected and suspended in 1 mL of diluted DCFH-DA at a cell concentration of 1 × 10^6^/mL and incubated for 20 min with 5% CO_2_ and at 37 °C. Cells were collected from each group, resuspended in 500 μL PBS, 1 × 10^6^/mL, and ROS was detected on the machine.

### Enzyme-linked Immunosorbent Assay (ELISA)

ELISA kits were used to detect the levels of IL-1β, TNF-α, and IL-6 in each group of cells. First, the standards were diluted, and a standard curve was constructed. The standard, blank, and sample wells were assembled on the enzyme standard plate. Next, 50 μL of standard was added at different concentrations, followed by 40 μL of sample and 10 μL of biotin-labeled antibodies. The plates were sealed and incubated at 37 °C for 30 min. Following washing, color developer was added to each well and developed at 37 °C for 10 min. Finally, 50 μL of termination solution was added to terminate the reaction, and the absorbance of each well was measured sequentially at 450 nm. All ELISA kits were purchased from Bioswamp (China).

### Tumor Formation in Nude Mice

Twenty-four strains of SPF male nude mice at 4 weeks old were selected. All mice were obtained from Hunan Slaughter Kingda. The mice were injected subcutaneously with A549 cells in the right proximal axilla with a concentration of 1 × 10^7^ cells/mL and a volume of 100 μL at 25 °C. The SIRT3 mimics group received one 2-h hypoxia treatment per day and was transfected with the SIRT3 overexpression vector. The Vector group received one 2-h hypoxia treatment per day and was also transfected with the SIRT3 overexpression vector. After the experimental cycle of 28 d, mice were anesthetized with 1% sodium pentobarbital, photographed, and tumor tissues were obtained.

### Immunohistochemistry

The fixed tumor tissues were dehydrated and embedded in wax according to the conventional procedure. The wax samples were cut into 3-μm slices. Antigen repair was performed at a high pressure (125 °C, 103 KPa). The slides were incubated in a 3% H_2_O_2_ wet box for 10 min to eliminate the endogenous peroxidase activity. The primary antibody SIRT3 (1:100) was then added dropwise, and the samples were incubated overnight at 4 °C. The Maxvision secondary antibody was added dropwise, and the samples were incubated for 30 min at 37 °C. DAB staining. Tissues were then re-stained for 3 min using hematoxylin, followed by alcohol fractionation with 1% hydrochloric acid. Tissues samples were observed under a microscope, and pictures were recorded. The relevant parts of the samples were collected and analyzed.

### Statistical Analysis

Data were analyzed using GraphPad Prism 8 software. The measurement data are presented as mean ± standard deviation (mean ± SD). Data comparison among multiple groups was performed using one-way ANOVA, and *P* < 0.05 indicated statistically significant differences.

## Results

### Effects of SIRT3 on Cell Proliferation and Migration Under Hypoxic Conditions

**Fig. 1:**
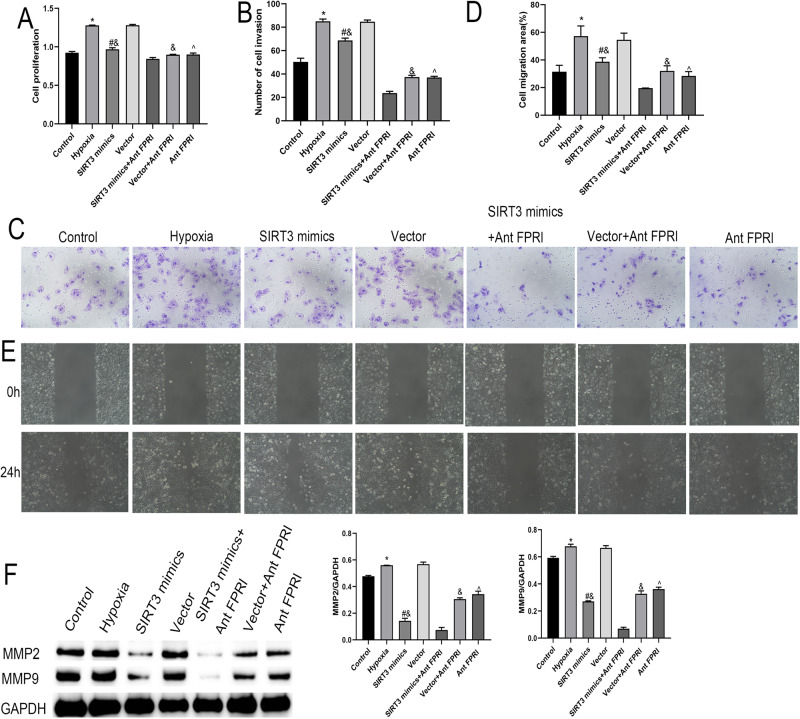
SIRT3 overexpression and FPRl low expression can inhibit tumor cell proliferation, invasion, and migration in a hypoxic environment. **A** MTT assay for cell proliferation. **B**, **C** Transwell assay detection of cell invasion. **D**, **E** Cell migration via cell scoring. **F** Western-blot detection of the expressions of migration-associated proteins MMP-2 and MMP-9. *Compared with Control group, *P* < 0.05; #Compared with Vector group, *P* < 0.05; &Compared with SIRT3 mimics+Ant FPRl group, *P* < 0.05; ^Compared with Hypoxia group, *P* < 0.05

Cell proliferation, invasion, and migration were significantly higher in the Hypoxia group than in the Control group, significantly lower in the Ant FPRl group than in the Hypoxia group, significantly lower in the SIRT3 mimics group than in the Vector group, and significantly lower in the SIRT3 mimics+Ant FPRl group than in the SIRT3 mimics and Ant FPRl groups (Fig. 1A–E). In addition, the expressions of MMP2 and MMP9 were significantly up-regulated in the Hypoxia group cells compared with the Control group, significantly down-regulated in cells of the Ant FPRl group compared with the Hypoxia group, significantly down-regulated in the SIRT3 mimics group compared with the Vector group, and down-regulated in cells of the SIRT3 mimics+Ant FPRl group compared with SIRT3 mimics and Ant FPRl groups (Fig. 1F). These results indicate that SIRT3 overexpression and FPRl low expression can inhibit tumor cell proliferation, invasion, and migration in a hypoxic environment.

### Effects of SIRT3 on Cellular Inflammation and ROS Levels Under Hypoxic Conditions

**Fig. 2:**
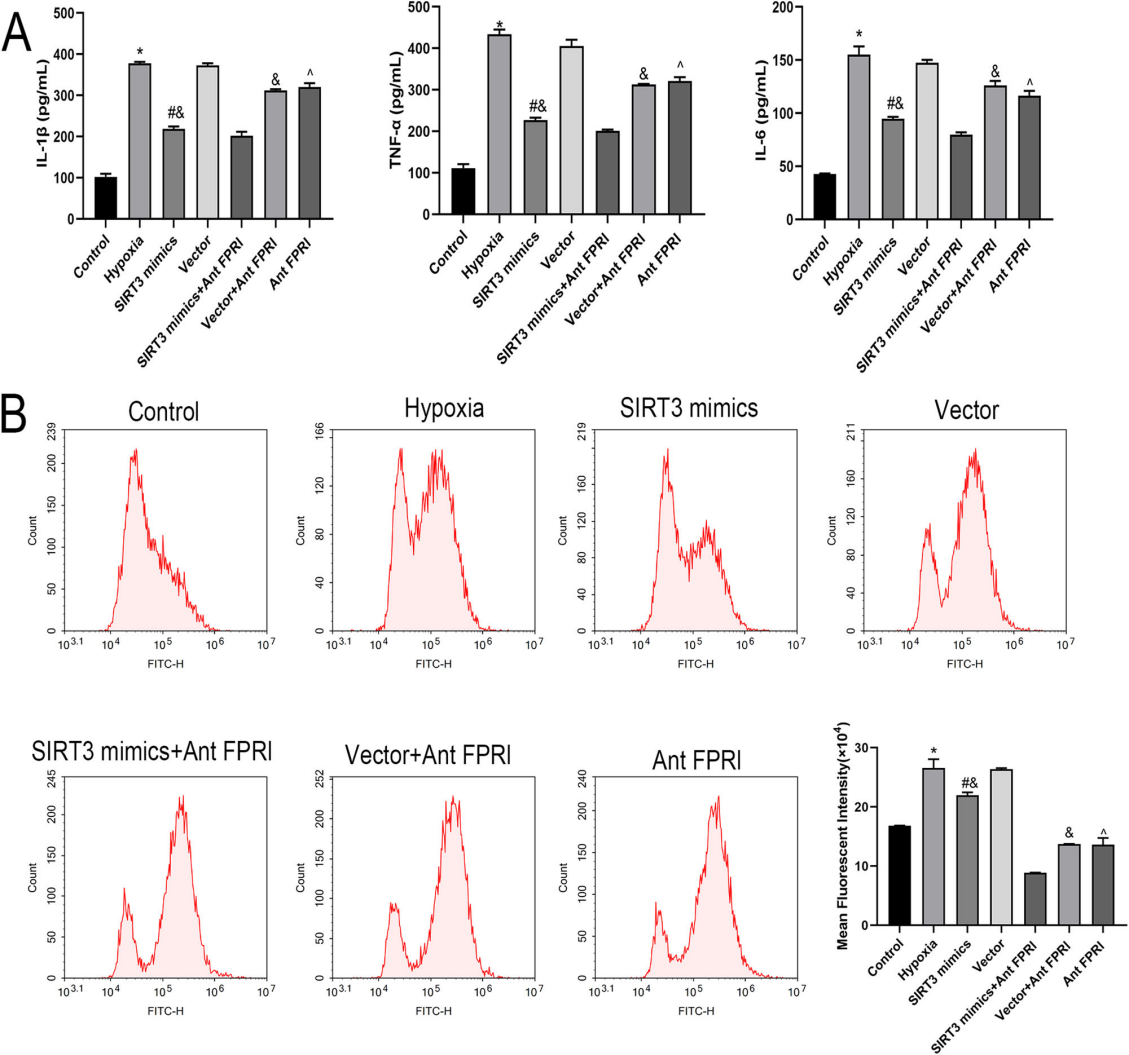
Effects of SIRT3 on cellular inflammation and ROS levels under hypoxic conditions. **A** ELISA detection of the expression levels of IL-1β, TNF-α, and IL-6 in each group of cells. **B** Flow cytometry detection of ROS levels in cells of each group. *Compared with Control group, *P* < 0.05; #Compared with Vector group, *P* < 0.05; &Compared with SIRT3 mimics+Ant FPRl group, *P* < 0.05; ^Compared with Hypoxia group, *P* < 0.05

The levels of IL-1β, TNF-α, IL-6, and ROS were significantly higher in cells of the Hypoxia group than in the Control group, significantly lower in cells of the Ant FPRl group than in the Hypoxia group, significantly lower in cells of the SIRT3 mimics group than in the Vector group, and significantly lower in cells of the SIRT3 mimics+Ant FPRl group than in the SIRT3 mimics and Ant FPRl groups (Fig. 2A, B).

### Effect of SIRT3 on FRP1/HIF-1α Axis Under Hypoxic Conditions

**Fig. 3:**
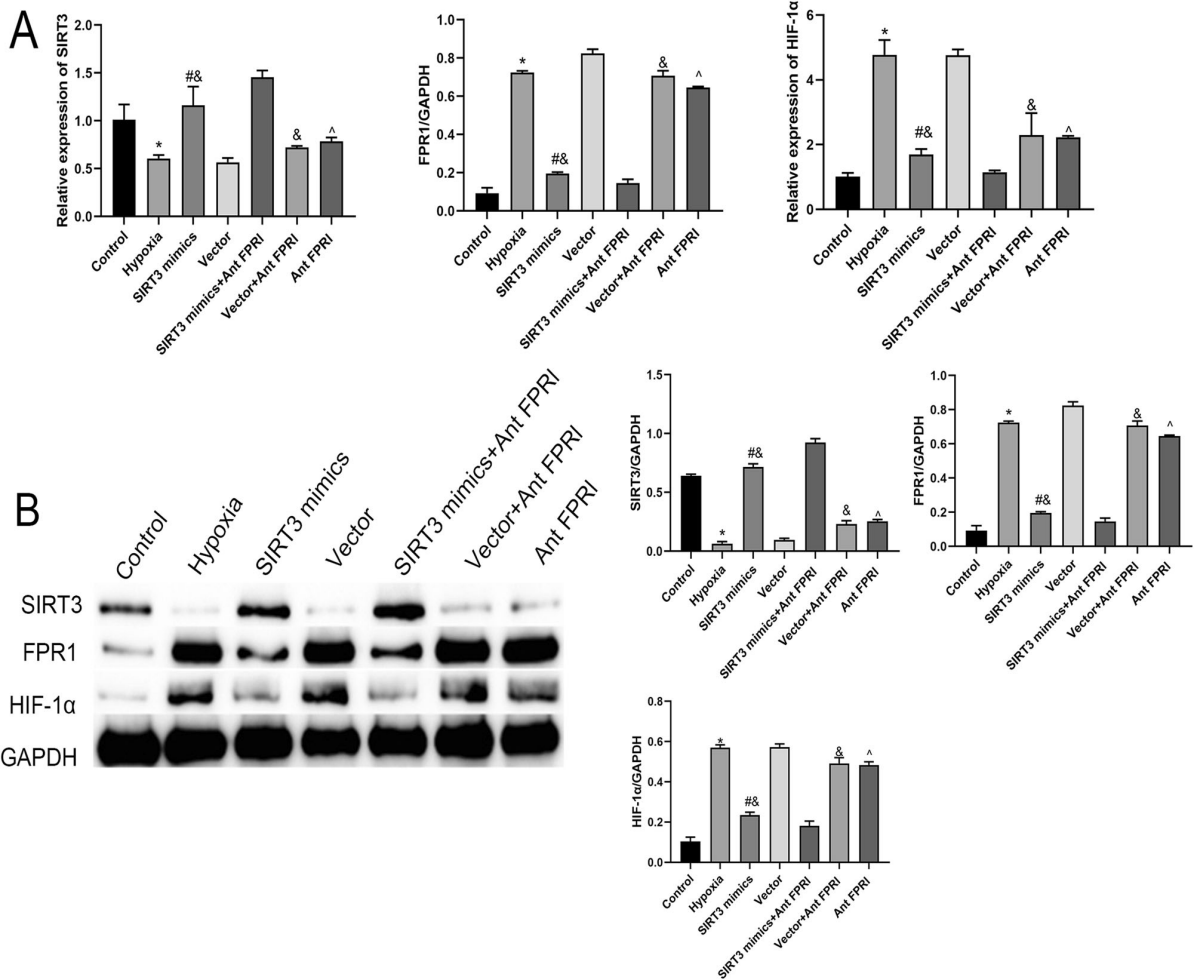
Effect of SIRT3 on FRP1/HIF-1α axis under hypoxic conditions. **A** qPCR detection of the mRNA expressions of SIRT3, FPR1, and HIF-1α in cells. **B** Western-blot detection of the protein expressions of SIRT3, FPR1, and HIF-1α in cells. *Compared with Control group, *P* < 0.05; #Compared with Vector group, *P* < 0.05; &Compared with SIRT3 mimics+Ant FPRl group, *P* < 0.05; ^Compared with Hypoxia group, *P* < 0.05

The mRNA and protein expression levels of SIRT3 were significantly down-regulated, and the mRNA and protein expression levels of FPR1 and HIF-1α were significantly up-regulated in cells of the Hypoxia group compared with the Control group. Both the mRNA and protein expression levels of SIRT3 were significantly up-regulated and those of FPR1 and HIF-1α were significantly down-regulated in cells of the Ant FPRl group compared with the Hypoxia group. Compared with the Vector group, both mRNA and protein expression levels of SIRT3 were significantly up-regulated in cells of the SIRT3 mimics group, whereas the mRNA and protein expression levels of FPR1 and HIF-1α were significantly down-regulated. When compared with the SIRT3 mimics and the Ant FPRl groups, both mRNA and protein expression levels of SIRT3 were significantly up-regulated, whereas the mRNA and protein expression levels of FPR1 and HIF-1α were significantly down-regulated in cells of the SIRT3 mimics+Ant FPRl group (Fig. 3A, B). Therefore, SIRT3 may inhibit the FRP1/HIF-1α axis.

### SIRT3 Inhibits Inflammation and Tumor Development in Nude Mice Under Hypoxic Conditions

**Fig. 4:**
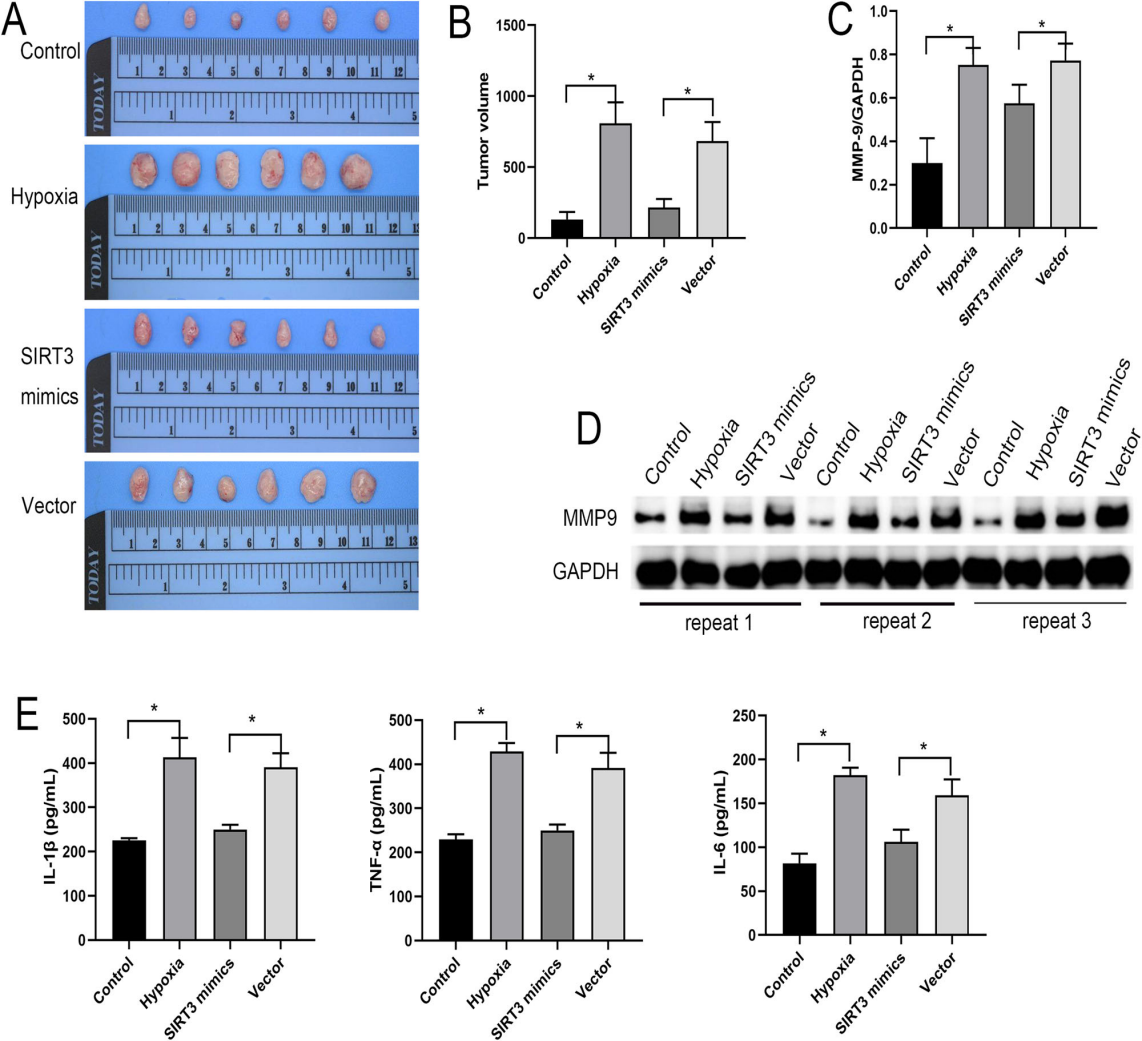
Inhibition of inflammation and tumor development in nude mice by SIRT3 under hypoxic conditions. **A**, **B** Detection of tumor volume in nude mice. **C**, **D** Western-blot assay detection of the expression level of MMP-9 in tumor tissues. **E** ELISA detection of the expression levels of IL-1β, TNF-α, and IL-6 in tumor tissues

We then examined the tumor formation assay in nude mice and found that the tumor size was significantly larger in the Hypoxia group than in the Control group. Tumor volume was significantly lower in the SIRT3 mimics group than in the Vector group (Fig. 4A, B). Moreover, the expressions of MMP9 were significantly up-regulated in the Hypoxia group compared with the Control group and significantly down-regulated in the SIRT3 mimics group compared with the Vector group (Fig. 4C, D). The levels of IL-1β, TNF-α, and IL-6 were significantly higher in the Hypoxia group than in the Control group and significantly lower in the SIRT3 mimics group than in the Vector group (Fig. 4E). These results suggest that SIRT3 overexpression can inhibit the development of tumors in nude mice.

### SIRT3 Affects Tumor Development in Nude Mice via FRP1/HIF-1α Axis Under Hypoxic Conditions

**Fig. 5:**
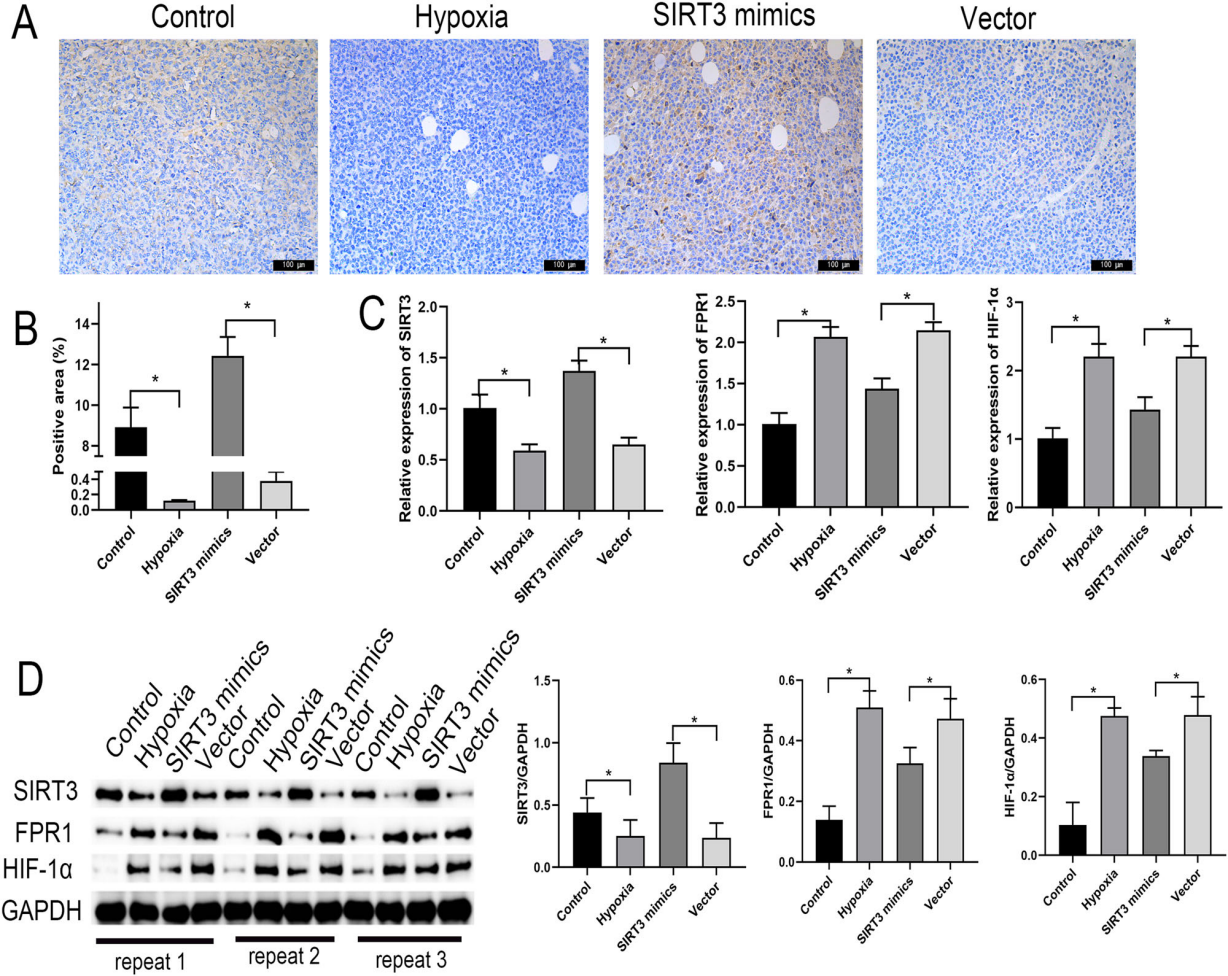
SIRT3 affects tumor development in nude mice under hypoxic conditions via the FRP1/HIF-1α axis. **A**, **B** Immunohistochemical detection of SIRT3 expression in tumor tissues. **C** qPCR detection of the mRNA expressions of SIRT3, FPR1, and HIF-1α in tumor tissues. **D** Western-blot detection of the protein expressions of SIRT3, FPR1, and HIF-1α in tumor tissues

The expressions of SIRT3 in tumor tissues were considerably lower in the Hypoxia group than in the Control group and significantly higher in the SIRT3 mimics group than in the Vector group (Fig. 5A, B). In addition, the mRNA and protein expression levels of SIRT3 were significantly down-regulated in tumor tissues in the Hypoxia group compared with the Control group. Compared with the Vector group, the mRNA and protein expression levels of SIRT3 were significantly up-regulated in tumor tissues of the SIRT3 mimics group.

## Discussion

In this study, we investigated the role of SIRT3 on tumor development in lung cancer cells and tumor-forming nude mice via the ROS-FPR1/HIF-1α axis in a hypoxic environment. Our results show that SIRT3 overexpression in a hypoxic environment inhibits cell proliferation, invasion, migration, and inflammation levels. Furthermore, we observed the same results in tumorigenic experiments in nude mice. Its related study mechanism confirms that SIRT3 may inhibit tumor cell progression via the ROS-FPR1/HIF-1α axis.

Tumor tissue hypoxia is a characteristic of solid tumors, and hypoxic cancer cells can accumulate lipids via metabolic changes, thus protecting cancer cells from oxidation and endoplasmic reticulum stress and playing an important role in promoting cancer cell proliferation following re-oxidation [12]. In previous studies, HIF-1α was found to be overexpressed in numerous human cancers and multiple cell types, and HIF-1α activity was associated with tumorigenicity and metastasis [13]. The expression of HIF-1α is a marker of hypoxia, and in a normoxic environment, the HIF-1α-expressed protein can be rapidly degraded in cells by prolyl hydroxylase, whereas in a hypoxic environment, this degradation pathway is disrupted. This results in the accumulation of HIF-1α in tumor cells, which ultimately enables the tumor to metabolize glucose to lactate and survive in a hypoxic environment [14]. In the present study, the expression of HIF-1α was significantly up-regulated in Hypoxia group cells; therefore, this hypoxic environment was successfully constructed.

As a tumor suppressor, SIRT3 can participate in various metabolic processes by deacetylating downstream protein substrates [15]. Its dysregulation can be involved in the development of several diseases. In this study, SIRT3 overexpression inhibited the proliferation, invasion, and migration of tumor cells under a hypoxic environment. The results were similar to those of previous studies. Zhang et al. [16] found that the expression of SIRT3 was significantly lower in hepatocellular carcinoma tissues than in normal liver and cirrhotic tissues. Xiao et al. [17] determined that SIRT3 expression showed a trend of down-regulation in lung adenocarcinoma cells compared to that for normal cells. SIRT3 increases the adaptive capacity of endothelial cells via deacetylation in hypoxic environments [18]. Cao et al. [19] found that elevated SIRT3 inhibited the viability, proliferation, and invasion of lung cancer cells. In different cancer pathological types, SIRT3 may trigger different key proteins and signaling pathways to initiate oncogenic or antitumor effects [20]. The deletion of SIRT3 enhances the activation of HIF-1α and hypoxia induces ROS production at the Q0 site of mitochondrial complex III [7]. In the present study, we found that the overexpression of SIRT3 in a hypoxic environment could significantly reduce the expressions of HIF-1α and ROS in cells, while decreasing the levels of inflammatory factors IL-1β, TNF-α, and IL-6.

FPR1 is involved in a wide range of physiopathological processes, such as cell migration, invasion, and tumorigenicity, in various tumor cells [10, 21, 22], and was found to promote cardiovascular disease progression [11]. In this study, the overexpression of SIRT3 significantly decreased the expression of FPR1 in tumor cells under a hypoxic environment. This result is consistent with the results obtained using the FPR1 antagonist. In addition, our previous studies have revealed that the expression levels of the FPR1 protein and mRNA increased following hypoxia, and inhibiting FPR1 activity prevented the effect of hypoxia on FPR1 expression [9]. Therefore, the expression of HIF-1α is a sign of hypoxia. In this experiment, the overexpression of SIRT3 significantly decreased the expressions of FPR1 and HIF-1α.

## Conclusion

This study found that the overexpression of SIRT3 in a hypoxic environment, both in vivo and in vitro, inhibited tumor cell proliferation, invasion, migration, ROS, and inflammation levels. The mechanism may be related to the regulation of the ROS-FPR1/HIF-1α axis by SIRT3.

## Data Availability

All data from this study can be requested directly from the corresponding author upon reasonable request.
